# Highlighted role of “IL17 signaling pathway” in gastroesophageal reflux disease 

**Published:** 2020

**Authors:** Mona Zamanian Azodi, Mohammadreza Razzaghi, Habib Malekpour, Majid Rezaei-Tavirani, Mostafa Rezaei-Tavirani, Mohammad Hossein Heidari

**Affiliations:** 1 *Proteomics Research Center, Shahid Beheshti University of Medical Sciences, Tehran, Iran*; 2 *Laser Application in Medical Sciences Research Center, Shahid Beheshti University of Medical Sciences, Tehran, Iran*; 3 *Gastroenterology and Liver Diseases Research Center, Research Institute for Gastroenterology and Liver Diseases, Shahid Beheshti University of Medical Sciences, Tehran, Iran*; 4 *Firoozabadi Hospital, Faculty of Medicine, Iran University of Medical Sciences, Tehran, Iran*; 5 *Proteomics Research Center, Faculty of Paramedical Sciences, Shahid Beheshti University of Medical Sciences, Tehran, Iran*

**Keywords:** Gastroesophageal reflux disease, Protein-protein interaction network analysis, Gene ontology, Biomarker

## Abstract

**Aim:**

The aim of this study is to assess the molecular profile of gastroesophageal reflux disease (GERD) via Protein-protein interaction (PPI) network analysis and gene ontology (GO) investigation.

**Background:**

GERD which affects the life of about 30% of people is associated with high costs in the human papulation. Several risk factors such as smoking, eating habits, BMI, and dysfunction of lower esophageal sphincter have been reported to contribute to the onset and progression of GERD. The roles of some types of interleukins and inflammatory factors as molecular features of GERD are investigated.

**Methods:**

Genes related to GERD were analyzed by Cytoscape v.3.7.2 and the corresponding plug-ins. ClueGO and CluePedia assessed the gene ontology and action type properties for the central nodes.

**Results:**

The results indicated that there are 12 hub-bottlenecks almost all of which except ALB are dispersed in the network clusters 1 and 2. Il17 signaling pathway among 7 identified biochemical pathways was also detected as a most related annotation for these central genes.

**Conclusion:**

Numbers of 11 critical genes and one pathway (IL17 signaling pathway) were highlighted as the deregulate genes and pathway in GERD. Common molecular features of GERD and cancer appeared.

## Introduction

 Gastroesophageal reflux as a common disease could be costly and cause reduction in the quality of life (1). Furthermore, it could be a precursor for development of the esophageal cancer (2). About 30% of adults are diagnosed with GERD in western countries. Life style factors are associated with the incidence of this disease including smoking, eating habits, BMI, and exercise (3-5). Further, dysfunction of anti-reflux barriers such as the lower esophageal sphincter is known to be involved in the pathogenicity of this disease (6). On the other hand, it has been reported that certain molecular factors also contribute to the incidence of this type of digestive system disease (7) such as some types of interleukins and inflammatory factors (8, 9). Molecules participate in biological processes that could be important in the disease underlying mechanisms. Genomics, proteomics, and metabolomics are the approaches that provide information related to the molecular profile of the corresponding disease. Bioinformatics can assist in obtaining additional data of molecules’ associations known as biomarkers in a disease profile. In this regard, the genes, proteins, and metabolites from high throughput studies could be studied more in depth through bioinformatics (10). Protein-protein interaction (PPI) network analysis is an approach for detecting topological features of a disease as an interactome. Interacted elements with higher centrality properties in the map could be valuable biomarkers. The central elements known as nodes can be identified by analyzing the key parameters of the corresponding network. Two important types of these parameters are degree and betweenness centrality (11). The central nodes or genes are essential for the network strength and function. The biomarker candidates with these characteristics are more promising in the disease pathogenicity (12). Genes could be retrieved through STRIG database search as Cytoscape application in this regard. These genes are introduced based on disease scores, showing their linkage to the disease. In this study, genes linked to gastroesophageal reflux disease (GERD) were analyzed and investigated by protein-protein interaction network approach to achieve a better insight into this disease. 

## Methods

“Gastroesophageal reflux disease” is the keyword searched in the STRING database source as a plug-in of Cytoscape software V.3.7.2 for *Homo sapiens* (13). The number of nodes for retrieving the most related nodes to the disease was set to the 100 nodes as the default setting. In addition, the cut-off score for the interaction between the nodes was 0.4. There are four query (protein search, metabolite query, disease query, and PubMed search) in STRING database. The disease search would also provide other information aside from just finding the genes related to the disease (14). An example of this information is the disease score. This feature shows that how much the studied gene is related to the disease. The next step is to screen the constructed network through other plug-ins available in the Cytoscape. Network Analyzer provides algorithms for investigating the network fundamental nodes in terms of centrality. Centrality is analyzed through certain parameters including degree and betweenness as popular ones. Those nodes with a high value of these parameters are considered as central genes. The node with high values of degree is recognized as hub, while the node with high values of betweenness is called bottleneck. The nodes with both features are called hub-bottlenecks which are very important in the network strength and stability (15). The next step is analyzing the other feature of the network, which is clustering. Genes within the high scored clusters have essential roles where some of the hub-bottlenecks are detectable in the complexes. These clusters could contribute to different processes and pathways. The software designed for this investigation is Molecular Complex Detection (MCODE V.1.5). This application finds densely interacting areas in the constructed network (16). Vertex weighting is the foundation of this analysis procedure. The highest scored genes in these complexes are called seed. The statistical criteria for this exploration are: degree cut-off; 2, node score cut off; 0.2, K-core; 2, and max depth; 100.

Gene ontology (biochemical pathways) and action relation were the next steps for analyzing the hub-bottleneck and seed nodes by ClueGO v.2.5.5 + CluePedia (17). The number of genes per term was considered 3 and percentage of genes per term was set 4 as default options for gene ontology analysis. The kappa score for term grouping was set as 0.5; for the action type analysis, different actions were chosen for the assessment. Also, the kappa cut-off score for this analysis which is principally between 0-1 was chosen 0.5 as medium score. The pathways were obtained from Kyoto Encyclopedia of Genes and Genomes (KEGG) database and Wiki Pathways database sources. 

## Results

**Figure 1:**
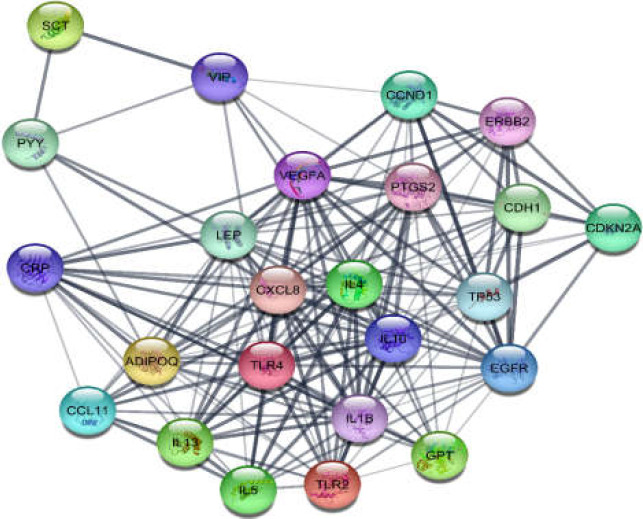
Cluster-1 including 24 nodes and 176 edges. ADIPOQ appeared as the seed node

**Figure 2:**
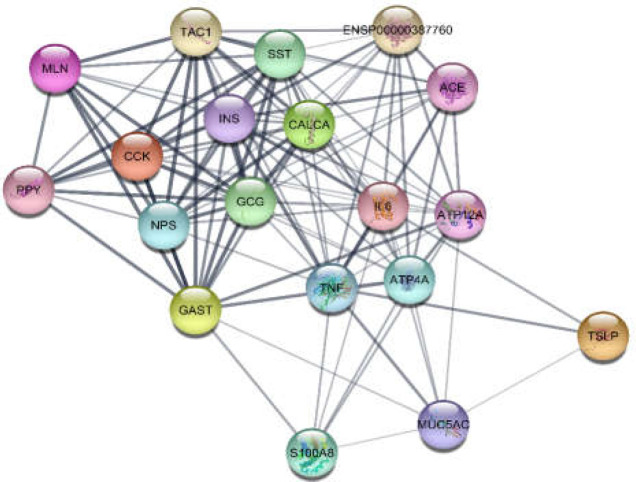
Cluster-2 including 19 nodes and 111 edges. MUC5AC was introduced as the seed node

The network of GERD was constructed by 100 nodes and 903 edges. The network contained a main connected component and 6 isolated nodes including SYMPK, CLASRP, PRB1, ZBED1, SLC22AA4, and EHD4 (the data not shown). Five protein clusters were obtained from the protein cluster analysis. Among them, the first top two (as clusters 1 and 2) are depicted in Figures 1 and 2. Cluster-1 has a score of 15 and Cluster-2 has a score of 12. The first ranked cluster contained 24 nodes and 176 edges, while the second cluster contained 19 nodes with 111 edges. ADIPOQ and MUC5AC were the seeds for the cluster-1 and 2, respectively. 

**Figure 3:**
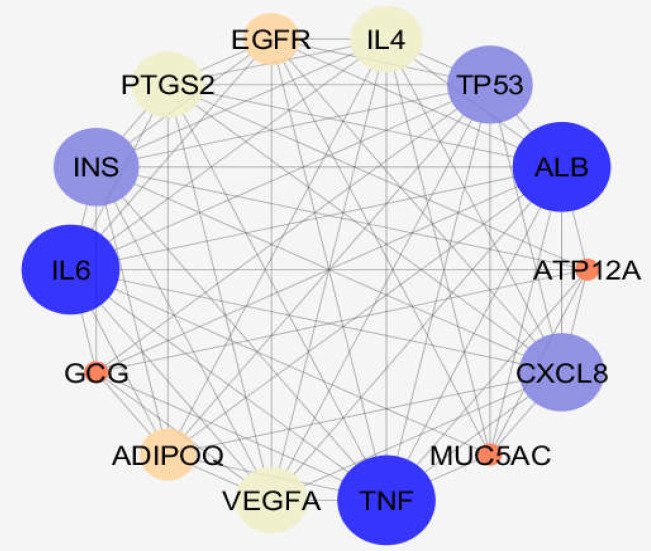
A sub-network including 12 hub-bottleneck nodes and two seeds of clusters 1 and 2. The nodes have been laid out based on degree value

Centrality analysis recognized some genes with important key participation in the network stability. These genes are recognized as hub-bottlenecks by using “Network Analyzer” application of Cytoscape software. About 20% of nodes based on degree value and betweenness centrality were selected as hubs and bottlenecks. The common genes with highest values of these two mentioned parameters (as hub-bottleneck nodes) are listed in Table 1 with their detailed information. A total of 12 genes were identified as hub-bottlenecks. From the topological analysis**, **it can be noted that none was from seed genes except for ALB. This gene was the highest scored hub-bottleneck and belonged to the third cluster. Most of the hub-bottlenecks were present in clusters 1 and 2. The connections between the hub-bottleneck nodes and the two seeds are shown in Figure 3.

**Figure 4:**
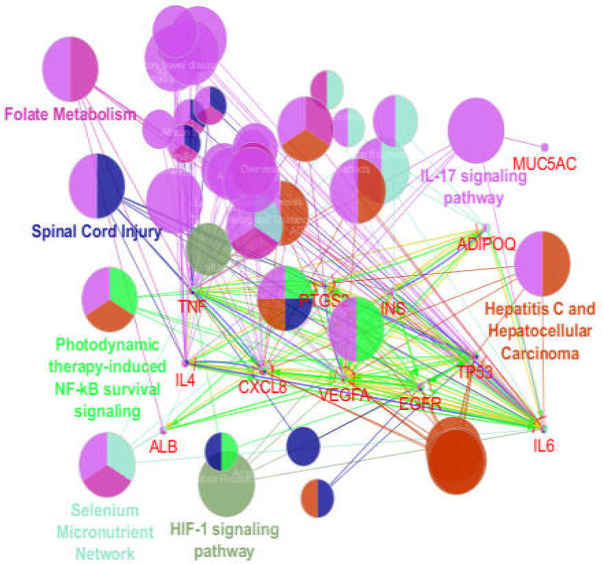
Biochemical pathway analysis of hub-bottlenecks plus seeds of clusters 1- 2 via ClueGO+CluePedia analysis. The terms are grouped in 7 clusters

**Table 1:** List of hub-bottlenecks nodes. The genes are ranked based on degree value. DN, DS, K, BC, and MC refer to disease name, disease score, degree, betweenness centrality, and MCODE Cluster, respectively

Row	DN	DS	K	BC	MC
1	ALB	1.9	59	0.1	Cluster 3
2	IL6	2	57	0.06	Cluster 2
3	INS	2	52	0.08	Cluster 2
4	CXCL8	2	51	0.05	Cluster 1
5	TNF	1.9	50	0.04	Cluster 2
6	PTGS2	2	41	0.05	Cluster 1
7	EGFR	1.5	40	0.03	Cluster 1
8	VEGFA	1.5	38	0.03	Cluster 1
9	IL4	1.7	38	0.02	Cluster 1
10	TP53	1.9	36	0.07	Cluster 1
11	GCG	1.8	33	0.02	Cluster 2
12	ATP12A	4	31	0.03	Cluster 2

**Figure 5:**
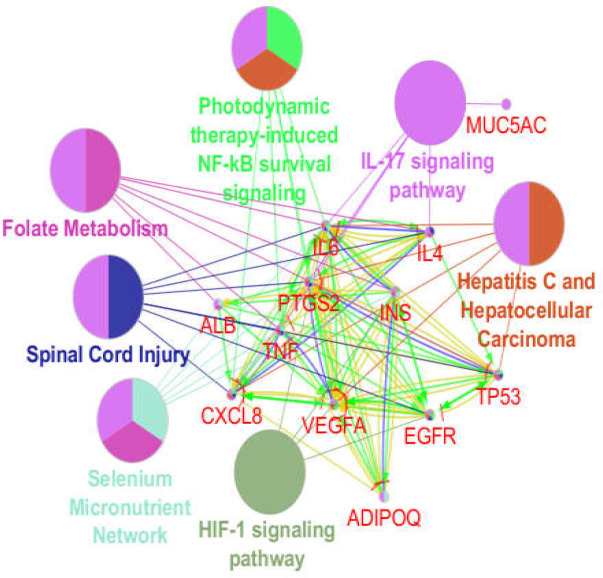
Results of biochemical pathway analysis of hub-bottlenecks plus seeds of cluster-1 and 2 via ClueGO+CluePedia analysis. For better resolution, the terms assigned as cluster header are shown while the other terms are not shown

**Figure 6:**
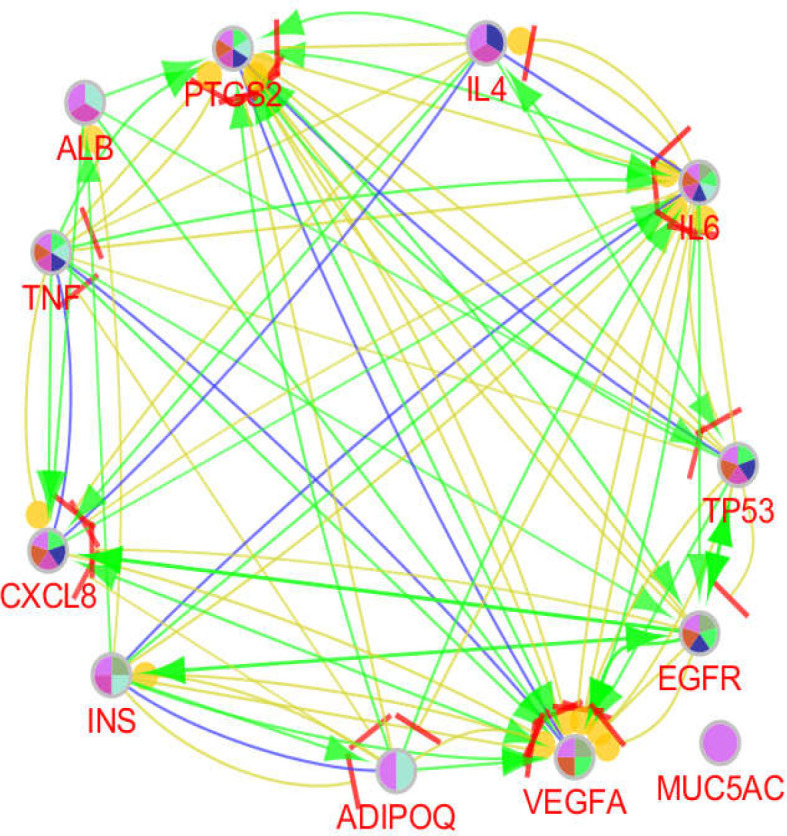
Action relationships including activation (green), inhibition (red), expression (yellow), and binding (blue) between the genes which were related to the determined biochemical pathways

**Figure 7:**
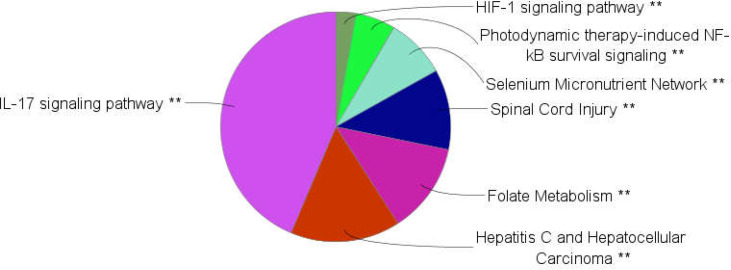
Clusters of biochemical pathways. Area refers to the frequency of terms in the cluster. **; it corresponds to the p≤0.01

Role of 14 critical elements including hub-bottlenecks and two seeds of clusters 1 and 2 was determined by ClueGO and CluePedia via pathway analysis, with the results shown in Figures 4-7. As depicted in Figure 4, ATP12A and GCG have not presented as related genes to the biochemical pathways.

## Discussion

The molecular study of different kinds of gastrointestinal diseases could assist in identifying the corresponding mechanisms of pathogenesis (11). 

One of them is gastroesophageal reflux disease (GERD) whose molecular analysis in terms of protein-protein interaction network exploration could provide further knowledge. Genes related to GERD were gathered by Cytoscape application, String disease query, after which topological analysis was performed. The constructed network showed that there are two main protein complexes in their pattern with some novel features. Further analysis for centrality properties of the network identified 12 hub-bottleneck genes dispersed mostly in clusters 1 and 2. Six of these central genes were contained in the cluster-1 and the rest were in other clusters especially cluster-2. AlB as the highlighted hub-bottleneck, however, was involved in the third cluster. Presence of hub-bottlenecks in cluster 1 and 2 indicates the importance of these two first ranked protein complexes. 

Further investigation revealed that there are condensed linkages between hub-bottlenecks and ADIPOQ and MUC5AC (the seeds for the cluster-1 and 2), though ATP12A and GCG lost about 40% of connections with the neighbors. These two hub-bottleneck nodes were not involved in the determined biochemical pathways. Thus, 7 clusters of 42 biochemical pathways were associated to the 10 hub-bottlenecks and 2 seed genes. Action role analysis of these 12 critical genes indicated that MUC5AC (the seed of cluster-2) has no action role. 

Pathway analysis is a suitable method to screen a set of proteins, genes, or metabolites (18). Pathway analysis showed that Il-17 signaling pathway is the main affected biochemical pathway in patients (see Figures 4 and 7). Based on the report of Z Xu et al., Il-23/Il-17 axis was upregulated in the mouse model of reflux esophagitis (19). As depicted in Figure 6, ALB, IL6, INS, CXCL8, TNF, PTGS2, EGFR, VEGFA, IL4, TP53, and ADIPOQ (10 hub-bottleneck nodes and one seed gene) are associated with Il-17 signaling pathway. 

The literature review of the introduced critical genes will provide useful and additional information about the molecular mechanism of GERD. PTGS2, known as Prostaglandin-endoperoxide synthase 2, or cyclooxygenase-2 or COX-2, appeared as a critical regulatory element in Figure 6. HR Ferguson et al. suggested that the COX-2 8473 C allele can be considered as a potential genetic biomarker for susceptibility to esophageal adenocarcinoma (20). 

Cxcl8 known as chemokine ligand 8 is the other important gene in the action map. It has been reported that CXCL8 and its cognate receptors facilitate the onset and progression of various types of cancers including melanoma, breast cancer, colorectal cancer, lung cancer, and prostate cancer (21). As displayed in Figure 6, Cxcl8 is activated by Il4, TNF, ALB, EGFR, and VEGFA. The hyper-activation of Cxcl8 could be associated with cancer promotion. GERD as a risk factor for laryngeal cancer was investigated by MA Qadeer et al. They reported that GERD is probably a risk factor for this type of cancer (22).

The highly activated and up-regulated gene in Figure 6 is IL6. Investigation of LS Wang et al. revealed that the serum level IL-6 in patients with esophageal squamous cell carcinoma was significantly higher than in healthy controls (23). It seems that more activation of IL6 which is associated with its upregulation in the case of GERD can be considered as a cancer risk factor.

EGFR and VEGFA are the two highly activated genes in Figure 6, while there are several suppressing vectors affecting VEGFA. S Bandla et al. reported that the level of EGFR and VEGFA changed in the two types of esophageal cancer; esophageal squamous cell carcinoma, predominant globally, and esophageal adenocarcinoma (24). This finding in line with other results indicates that GERD can be considered as a risk factor for cancer promotion.

## Conclusion

Our analysis showed that deregulation of ALB, IL6, INS, CXCL8, TNF, PTGS2, EGFR, VEGFA, IL4, TP53, and ADIPOQ is a major event in GERD which is associated with alteration in the several terms clustered as “IL17 signaling pathway”. The finding refers to common features of molecular mechanism that promotes both GERD and cancer.
